# Factors influencing general practitioners’ decisions about cardiovascular disease risk reassessment: findings from experimental and interview studies

**DOI:** 10.1186/s12875-016-0499-7

**Published:** 2016-08-05

**Authors:** Shannon McKinn, Carissa Bonner, Jesse Jansen, Armando Teixeira-Pinto, Matthew So, Les Irwig, Jenny Doust, Paul Glasziou, Kirsten McCaffery

**Affiliations:** 1 Screening and Test Evaluation Program (STEP), Sydney School of Public Health, The University of Sydney, Sydney, NSW Australia; 2 Centre for Medical Psychology & Evidence-based Decision-making (CeMPED), The University of Sydney, Sydney, NSW Australia; 3 Faculty of Health Sciences and Medicine, Bond University, Robina, QLD Australia

**Keywords:** Cardiovascular disease, Primary care, General practice, Prevention, Risk assessment

## Abstract

**Background:**

Guidelines on cardiovascular disease (CVD) risk reassessment intervals are unclear, potentially leading to detrimental practice variation: too frequent can result in overtreatment and greater strain on the healthcare system; too infrequent could result in the neglect of high risk patients who require medication. This study aimed to understand the different factors that general practitioners (GPs) consider when deciding on the reassessment interval for patients previously assessed for primary CVD risk.

**Methods:**

This paper combines quantitative and qualitative data regarding reassessment intervals from two separate studies of CVD risk management. Experimental study: 144 Australian GPs viewed a random selection of hypothetical cases via a paper-based questionnaire, in which blood pressure, cholesterol and 5-year absolute risk (AR) were systematically varied to appear lower or higher. GPs were asked how they would manage each case, including an open-ended response for when they would reassess the patient. Interview study: Semi-structured interviews were conducted with a purposive sample of 25 Australian GPs, recruited separately from the GPs in the experimental study. Transcribed audio-recordings were thematically coded, using the Framework Analysis method.

**Results:**

Experiment: GPs stated that they would reassess the majority of patients across all absolute risk categories in 6 months or less (low AR = 52 % [CI_95%_ = 47–57 %], moderate AR = 82 % [CI_95%_ = 76–86 %], high AR = 87 % [CI_95%_ = 82–90 %], total = 71 % [CI_95%_ = 67–75 %]), with 48 % (CI_95%_ = 43–53 %) of patients reassessed in under 3 months. The majority (75 % [CI_95%_ = 70–79 %]) of patients with low-moderate AR (≤15 %) and an elevated risk factor would be reassessed in under 6 months.

Interviews: GPs identified different functions for reassessment and risk factor monitoring, which affected recommended intervals. These included perceived psychosocial benefits to patients, preparing the patient for medication, and identifying barriers to lifestyle change and medication adherence. Reassessment and monitoring intervals were driven by patient motivation to change lifestyle, patient demand, individual risk factors, and GP attitudes.

**Conclusions:**

There is substantial variation in reassessment intervals for patients with the same risk profile. This suggests that GPs are not following reassessment recommendations in the Australian guidelines. The use of shorter intervals for low-moderate AR contradicts research on optimal monitoring intervals, and may result in unnecessary costs and over-treatment.

**Electronic supplementary material:**

The online version of this article (doi:10.1186/s12875-016-0499-7) contains supplementary material, which is available to authorized users.

## Background

International guidelines for cardiovascular disease (CVD) prevention recommend the use of absolute risk (AR) assessment to guide preventive medication, rather than treating blood pressure and cholesterol as individual risk factors [1–3]. Australian guidelines use age, sex, smoking, diabetes, systolic blood pressure and cholesterol ratio to estimate the risk of a cardiovascular event in the next 5 years, based on the Framingham model [1, 2, 4]. Preventive medication is recommended if AR is higher than 15 %, or 10–15 % with other risk factors (e.g. significant family history of CVD). Reassessment of AR is recommended after 2 years for low risk patients (<10 %), 6–12 months for patients at moderate risk (10–15 %), and according to clinical context for high risk patients (>15 %). Patients on medication or recommended to make lifestyle changes may be monitored more frequently, but AR does not need to be reassessed. These reassessment recommendations are based on consensus-based expert clinical judgement and the published literature [1, 2]. Other international guidelines use a 10 year timeframe, with varying medication thresholds and reassessment recommendations, if reassessment is addressed at all [5–8].

Previous studies have shown that general practitioners (GPs) do not consistently base their decisions regarding primary CVD prevention on AR [9–13]. The lack of uniformity in guideline recommendations for reassessment intervals, combined with the low utilisation of AR in practice is likely to result in highly variable reassessment. This is a potential concern, as reassessments that are too frequent can result in overtreatment and greater strain on the healthcare system [6, 14], while reassessments that are too infrequent could result in the neglect of high risk patients who require medication. This study aims to understand the different clinical and psychosocial factors that GPs consider when recommending the frequency of CVD risk reassessment and monitoring in primary CVD prevention.

## Methods

This study combines quantitative and qualitative data. Questions on reassessment intervals were presented to Australian GPs in two separate studies of CVD risk management [9, 12, 15, 16]. In this context, reassessment refers to a patient returning to the GP for specific reassessment of CVD risk, including re-measurement of blood pressure and/or cholesterol. Primary assessment and management results have been presented elsewhere [9, 12, 15, 16]. Ethical approval was obtained from the University of Sydney Human Research Ethics Committee (11–2011/14379) and the Sydney Local Health District Human Research Ethics Committee (Protocol No X11-0200 and HREC/11/RPAH/294), and informed consent was obtained from all participants.

### Study 1

GPs currently practicing in Australia were recruited between May and November 2012 at four GP conferences (see Table 1).

**Table 1:** GP characteristics – study 1

		Study 1 (*n* = 144)
Sex	M	62 (43 %)
	F	82 (57 %)
Age	<40	18 (13 %)
	40–49	33 (23 %)
	50–59	61 (42 %)
	60+	30 (21 %)
Years of practice	< 10	6 (7 %)
	10–19	23 (16 %)
	20–29	49 (34 %)
	30+	60 (42 %)
Number of GPs in practice	1–5	72 (50 %)
	6–10	44 (31 %)
	11–15	18 (13 %)
	16+	6 (4 %)
	Not applicable (Locum, etc.) or no answer	4 (3 %)
Self-reported use of AR (in practice)^a^	1 (Never)	18 (13 %)
	2	27 (19 %)
	3	39 (27 %)
	4	38 (26 %)
	5 (Always)	21 (14 %)
Self-reported use of AR (in study cases)^a^	1 (Never)	12 (8 %)
	2	15 (10 %)
	3	37 (26 %)
	4	45 (31 %)
	5 (Always)	34 (24 %)

^a^1–5 Likert scale (1 = never, 5 = always); Percentages may not always add up to 100 due to missing responses

In a paper-based questionnaire, respondents viewed a generic patient scenario followed by a table with the relevant values for AR, systolic blood pressure, total cholesterol/HDL ratio, HDL, total cholesterol, age, gender and smoking status. Four sets of cases were developed (see Table 2).

**Table 2:** Patient case descriptions – study 1

Generic patient^a^ scenario	Description	
	A regular patient of yours presents for a “check-up” and has no current symptoms. He/she has been trying to improve their diet and increase their physical activity levels. You have several previous blood pressure readings at approximately the same level as observed today. A recent test of electrolytes, liver function and renal function was normal.	
	• BMI: 27	
	• Past medical history: nil of note	
	• Family history: mother died of bowel cancer, nil family history of ischaemic heart disease	
	• Social history: married, lives at home	
	• Ethnicity: Caucasian	
**Patient case category**	**Individual risk (IR)**	**Absolute Risk (IR)**
A (high IR, lower AR)	High (either blood pressure or cholesterol)	Lower (≤15 %)
Ai	High (blood pressure only – systolic blood pressure [SBP] ≥147 mmHg	Lower (≤15 %)
Aii	High (cholesterol only – total cholesterol/HDL ratio [TC/HDL] ≥6.5 mmol/L	Lower (≤15 %)
B (high IR, high AR)	High (SBP ≥147 mmHg, TC/HDL ≥6.5 mmol/L)	High (>15 %)
C (lower IR, high AR)	Lower (SBP <147 mmHg, TC/HDL <6.5 mmol/L)	High (>15 %)
D (lower IR, lower AR)	Lower (SBP <147 mmHg, TC/HDL <6.5 mmol/L)	Lower (≤15 %)

^a^Applies to all patient case categories

Each GP viewed 11 randomly selected cases, from a pool of 43. GPs were asked: whether they would prescribe cholesterol medication and/or blood pressure medication (yes/no for each); when they would reassess (open ended); and could comment on the case (open ended). A more detailed explanation of the method and cases is described elsewhere [12].

### Statistical analysis

To analyse the GPs’ decisions regarding time to reassessment according to risk profile, taking into account case clustering per GP, we used logistic regressions, fitted through generalised estimation equations (GEEs). The GEEs will produce similar estimates to the conventional logistic regression but the confidence intervals will be more conservative because of the correlation between observations due to clustering [17]. Time to reassessment was dichotomised: <6 months and ≥6 months. 95 % CIs for percentages were estimated from the GEEs, and statistical analysis was conducted using SPSS V.21.

### Study 2

Semi-structured interviews covering CVD risk assessment and management were conducted with 25 GPs between October 2011 and May 2012 [9] (see Table 3 for participant characteristics). GPs were recruited via a letter of invitation through their Divisions of General Practice. Participants were asked to describe how they would manage three hypothetical cases (see Table 4), and were asked specific questions about how they reassess primary CVD risk (see Additional file 1). Interviews were conducted via telephone (*n* = 23) or face to face (*n* = 2) by two public health researchers (SM, CB), and were audio recorded and transcribed verbatim. Interviews lasted between 22 and 55 min. A framework analysis method was used [18]. Three authors (SM, CB, JJ) developed the coding framework by independently coding a subset of the data, and discussing emerging themes to develop a preliminary coding scheme, which was discussed and reviewed with an experienced qualitative researcher (KM) to develop the coding framework. SM and CB coded the remaining transcripts, with new themes and revisions to the coding framework discussed throughout the process. SM examined the final framework in order to identify overarching themes and relationships, and results were discussed with all authors, including two academic GPs (JD, PG).

**Table 3:** GP Characteristics – study 2

		Study 2 (*n* = 25)
Sex	M	10 (40 %)
	F	15 (60 %)
Age	< 40	6 (24 %)
	40–49	8 (32 %)
	50–59	7 (28 %)
	60+	4 (16 %)
Years of practice	< 10	5 (20 %)
	10–19	6 (24 %)
	20–29	9 (36 %)
	30+	5 (20 %)
Number of GPs in practice	1–5	10 (40 %)
	6–10	13 (52 %)
	11–15	2 (8 %)

**Table 4:** Patient case descriptions – study 2

Case	Sex	Age	Smoking status Y/N	SBP (mmHg)	TC (mmol/L)	HDL (mmol/L)	TC/HDL	AR^a^ (%)	Study 1 equivalent case category
1	Male	61	N	156	4.9	1.6	3	9	Ai
2	Male	61	N	116	6.4	1	6.4	9	Aii
3	Male	61	Y	131	5.4	1.2	4.5	16	C

^a^Absolute risk was not reported systematically to all GPs. GPs were informed of the patients’ AR if they asked for it, or if they had previously mentioned using AR in their practice

## Results

### Study 1

Table 5 shows time to reassessment according to AR categories. Across all risk categories, the majority of patients (71 %, CI_95%_ = 67–75 %) would be recalled for reassessment in under six months. The high and moderate AR categories had similar reassessment intention within 6 months (87 %, CI_95%_ = 82–90 % and 82 % CI_95%_ = 76–86 %, respectively), while only 52 % (CI_95%_ = 47–57 %) of the patients with low AR would be reassessed in this time frame, as reported by the GPs. Overall, 48 % (CI_95%_ = 43–53 %) of patients would be recalled in under 3 months.

**Table 5:** Time to reassessment according to the absolute CVD Risk categories

		Absolute risk category (% of cases)		
	N	High (>15 %) *n* = 519	Moderate (10–15 %) *n* = 430	Low (<10 %) *n* = 635
Time to reassessment				
Less than 1 month	261	27	16	11
1 to <3 months	451	35	37	22
3 to <6 months	346	25	28	19
6 to <12 months	247	9	15	24
12 to <24 months	148	4	3	19
24 or more months	32	0	1	5

Many of the cases reassessed in under 6 months fall into category A (high IR/lower AR – either SBP ≥147 mm Hg or TC/HDL ≥6.5 mmol/L, AR ≤15 %). Seventy-five percent (CI_95%_ = 70–79 %) of patients in this category would be reassessed in less than 6 months. The majority of these cases fall into category Ai (high IR/lower AR – high blood pressure only: SBP ≥147 mm Hg, AR ≤15 %), with 90 % (CI_95%_ = 85–94 %) of patients reassessed in less than 6 months, compared to 59 % (CI_95%_ = 52–66 %) of patients in category Aii (high IR/lower AR – high cholesterol only: TC/HDL ≥6.5 mmol/L, AR ≤15 %). Table 6 shows time to reassessment according to case category.

**Table 6:** Time to reassessment according to patient case categories

	Patient case category (% of cases)					
	A	Ai	Aii	B	C	D
	High IR^a^ lower AR^b^ (combined)	High IR lower AR (BP only^c^)	High IR Lower AR (chol. only^d^)	High IR High AR^e^	Lower IR High AR	Lower IR Lower AR
	*N* = 788	*N* = 398	*N* = 390	*N* = 214	*N* = 277	*N* = 206
Time to reassessment						
< 1 month	17	29	4	45	11	1
1 to <3 months	34	47	21	38	34	4
3 to <6 months	24	14	35	15	34	15
6 to <12 months	18	8	28	1	14	31
12 to <24 months	6	1	10	1	7	39
24 or more months	1	1	2	0	0	10

^a^High IR: elevated individual risk factor

^b^lower AR: low or moderate absolute risk (≤15 %)

^c^BP only: systolic blood pressure ≥147 mmHg

^d^cholesterol only: total cholesterol/HDL ratio ≥6.5 mmol/L

^e^High AR: >15 %

In open-ended comments, GPs often referred to reassessment and monitoring of lifestyle more generally, particularly when preparing the patient for the possibility of starting medication. This was common for category B (high IR/high AR – SBP ≥147 mm Hg, TC/HDL ratio ≥6.5 mmol/L, AR >15 %) and category Ai (high IR/lower AR – high blood pressure only: SBP ≥147 mm Hg, AR ≤15 %) cases where reassessment in less than 6 months was recommended. Patients were also recalled for non-CVD related matters, such as cancer screening, post-menopausal health checks, and other blood tests to check organ function and blood count.

There was some confusion about using AR-based CVD prevention guidelines, and the concept of AR; several comments asked “where does the risk come from?” or asked why the patient’s AR was a particular level. This type of comment was made for cases in category C (lower IR/high AR – SBP <147 mm Hg, TC/HDL <6.5 mmol/L, AR >15 %) which could reflect a lack of understanding that the presence of multiple lower-level individual risk factors can amount to high AR.

### Study 2

Qualitative interviews provided insight into the range of reassessment times seen in the experimental study, with GPs identifying various functions for reassessment and monitoring. Table 7 provides a summary of reasons for shorter (<6 months) and longer (≥6 months) intervals. Although GPs were not specifically asked about monitoring function and intervals, these emerged as key themes in the qualitative analysis. In this context, monitoring refers to checks with the GP in regard to CVD medication, individual CVD risk factors, lifestyle changes, or other medical issues relevant for CVD (risk).

**Table 7:** Summary of reasons given by GPs for reassessment and monitoring intervals

Shorter monitoring and/or reassessment period (<6	Longer monitoring and/or reassessment period (6+ months)
**Patient drivers** • Low patient motivation to make lifestyle changes – may need to start medication earlier. • High patient motivation to make lifestyle changes – maintain motivation with good results. • Patient desires frequent monitoring and reassessment **Risk factor drivers** • Borderline for treatment with medication • Monitoring of blood pressure and cholesterol after lifestyle change prescription • Comorbidities • Weight monitoring • Smokers: frequent monitoring of other risk factors and opportunities to reassess willingness to quit. **GP drivers** • Strong focus on prevention/screening • Strong focus on reducing risk through lifestyle change rather than medication • View that you can monitor more often than recommended by guidelines without over-servicing	**Patient drivers** • High patient motivation - patient is motivated by early success with lifestyle change and can manage alone for longer intervals. • Longer time period needed for lifestyle changes to show results, in order not to discourage or demotivate patient with lack of success. **Risk factor drivers** • Risk factors have decreased or are well controlled with medication • No urgent CVD risk factors that need to be addressed, and/or absolute risk is low **GP drivers** • Too busy to reassess CVD risk unless requested by patient • Use of opportunistic monitoring and reassessment • Follow guideline recommendations on reassessment and monitoring frequency for low/moderate risk patients • Concern about over-servicing asymptomatic patients • Involvement of other practitioners in patient’s care (dietician, exercise physiologist, etc.)

### Reassessment and monitoring function

#### Psychosocial benefits

GPs made statements about the perceived psychosocial benefits of monitoring for their patients, especially for lifestyle change and weight management. GPs saw frequent monitoring as important for patient motivation, engagement and reassurance.

*“To keep them engaged…because if they are engaged with you and want to work on it you can do lots of things but if they disappear…you can’t do anything until they come.” (ID5)*

Reassessment of AR may play a part in this if the GP feels it may aid patient management, or that a change in AR will motivate the patient to continue with lifestyle management.

*“If you bring them back and you start saying ’ah look your cholesterol has come down, look at your risk, look on the CV calculator remember it was there, look where it is now’…once you start seeing a difference usually you’ll notice they get quite excited.” (ID22)*

#### Preparing the patient for medication

GPs saw reassessment of risk factors and regular monitoring of lifestyle changes as a method of getting patients accustomed to the idea that they may need to start potentially life-long medication.

*“If they’re not fine then…also get them used to the fact that they may well have to go onto medication so when they do go onto medication whether that be statins or whether it be anti-hypertensive, that they know that....that they do require, this wasn’t a once off.” (ID7)*

This approach may also involve reassessment of AR as a way of showing patients an objective measure of why they need to start medication.

*“I do find it useful to do the calculation again…for the patient so they can see there is some sort of objective reason why a medication would now be beneficial. They’ve…tried certain lifestyle changes in the community…and if they still haven’t reached target…it seems very reasonable then and to set some pharmacotherapy for that to be done.” (ID16)*

#### Identification of barriers

Another function of monitoring mentioned by GPs is to provide an opportunity to discuss barriers to adherence, for both lifestyle change and medication.

*“For those that aren’t getting there you may need to explore further…so you need to sort of be constructive and go back to if you haven’t lost weight well it’s not just come back in a month its well what are the barriers that are preventing this and how can we overcome those barriers.” (ID31)*

### Reassessment and monitoring intervals

#### Patient drivers: motivation to make lifestyle changes

Patient motivation also impacted monitoring and reassessment intervals, with frequent monitoring seen to be discouraging if the patient does not see the results of their efforts.

*“There is a fine line between doing it too early and them not seeing any results or…doing it…so they can see something happening.” (ID11)*

GPs cited both high and low patient motivation to change lifestyle as a reason to monitor frequently: for high motivation patients because it helps maintain motivation, and low motivation patients because they will need to commence medication sooner.

High motivation: *“if they....looked as though they were listening and trying to think ok I am going to modify my diet and lifestyle. I would say look lets test it in 2 months and see what change it makes and see if that is significant for you.” (ID29)*

Low motivation: *“the ones who really haven’t done anything, still putting on weight and smoking, I do try to see them more often.” (ID9)*

However, GPs also cited high and low patient motivation as a reason for longer monitoring and reassessment intervals: highly motivated patients may be monitored less frequently once they start achieving lifestyle change goals, while frequent monitoring of patients unwilling to make lifestyle changes or take medication may be seen as inappropriate and futile.

High motivation: *“Well I suppose if you see them 3 monthly first of all and they’ve done really well and they have dropped the weight and they’re exercising and they’re feeling really motivated then there is no point in checking their cholesterol again every 3 months.” (ID9)*

Low motivation: *“He is new to me today this guy and he has come in with cholesterol results basically every 3 months, they’ve been doing them for the last 2 years and I go what for, he has not changed over that time, he is not prepared to change and he is not going to take a statin and I go well what’s the point of doing it every 3 months, so we can have the same conversation.“(ID11)*

#### Patient drivers: demand for reassessment

Other patient driven factors mentioned by GPs that are not related to lifestyle change motivation include patient desire for regular blood tests, whether the GP thinks it is required or not:

*“I get patients who are very keen to do blood tests. Everybody seems very keen to know their cholesterol and their sugars and things…I sometimes have a battle with some patients because they want to do it* (cholesterol testing) *every 6 months, once a year is enough.” (ID10)*

Several GPs also mentioned gender as an element that influences monitoring and reassessment frequency, with men failing to present according to the schedule set by the GP.

*“If they are women they are going to come back, if they are men you to have to sort of chase them.” (ID5)*

#### Risk factor drivers

The presence of certain risk factors drove some GPs to recall patients more frequently, including comorbidities, and factors that are not taken into account in the AR calculation, such as weight and family history. Smokers who are not attempting to quit may also be encouraged by their GP to visit more frequently to monitor their other risk factors.

*“If you’ve got some severely obese kind of young guy with a massive family history you would be more concerned about him and more likely to chase him up than someone who is relatively fit and healthy and has the same absolute score.” (ID9)*

#### GP drivers

GPs’ own attitudes regarding prevention and guidelines influenced the frequency of monitoring, ranging from a strong focus on screening all patients, to concern about over-servicing.

*“I am measuring all those factors at least every 12 months on virtually all my patients…or maybe 6 months…that’s the way I run my practice, I do a lot of screening.” (ID26)*

*“I try to get them back at the 6 weeks, month, 6 weeks - 3 months, I know it’s always sooner than what the GP guidelines are but I like to keep it a little bit on the front burner while not massively over-servicing” (ID2)*

Some GPs were concerned about over-servicing asymptomatic patients. GPs mentioned that the consultation should be valuable to the patient, rather than an inconvenience.

*“I always feel really…hesitant about getting well people to come in and we don’t bulk bill, so getting them to come in and pay for a check which you know kind of says they’re ok.” (ID29)*

*“I don’t want the practice consultation to be viewed as a chore but really something....that they get meaning and get use out of it” (ID16)*

GP attitudes regarding lifestyle change also affected monitoring and reassessment intervals. Several GPs thought that more frequent monitoring should be encouraged for patients attempting lifestyle change.

*“If somebody has got a weight problem, obesity, then I will ask them to see me in a couple of weeks so I can check their weight and that motivates them. Because if they know they’re going to the Doctor and he’s going to check their weight they’re more conscious about weight…I won’t ask them to come and see me in say 3 or 6 months, they won’t come, we will lose them.” (ID38)*

## Discussion

The results of both studies show a substantial range in reassessment intervals for patients in the same CVD risk categories, with the interview study also revealing a wide range in GPs’ reasoning behind reassessment and monitoring intervals that is sometimes contradictory.

The experimental study results showed that across all risk categories, the majority of patients would be recalled for reassessment in under 6 months, and almost half in under 3 months. While high risk patients were reassessed in accordance with Australian guidelines, low-moderate risk patients were reassessed earlier [1, 2]. Perhaps of most concern is the finding that 95 % of low risk patients (AR <10 %) would be reassessed earlier that the Australian guidelines recommend [1, 2]; and 52 % of those would be reassessed within 6 months. In contrast, recent modelling research has found that reassessment of primary CVD risk before 8–10 years is not warranted for most people who do not require preventive medication at baseline [6].

The difference in reassessment times between category Ai cases (high IR/lower AR – high blood pressure only: SBP ≥ 147 mm Hg, AR ≤15 %) and category Aii cases (high IR/lower AR – cholesterol only: TC/HDL ≥ 6.5 mmol/L, AR ≤15 %) is consistent with previously reported results from this study which found that GPs’ decision-making was more influenced by individual risk factors than AR, especially for blood pressure lowering medication. Blood pressure lowering medication was prescribed in 83 % of the category Ai cases (high IR/lower AR – high blood pressure only: SBP ≥147 mm Hg, AR ≤15 %), compared with cholesterol lowering medication being prescribed in 34 % of the category Aii cases (high IR/lower AR – cholesterol only: TC/HDL] ≥6.5 mmol/L, AR ≤15 %) [12]. This pattern is confirmed in the reassessment intervals for these categories, with 90 % of high SBP/lower AR cases being reassessed in less than 6 months, compared to 59 % of high cholesterol/lower AR cases.

The broad range of reassessment times given by different GPs for the same cases is also suggestive of a general lack of awareness of the primary CVD prevention guidelines, in particular the use of AR. For example, one case in category Ai (high IR/lower AR – high blood pressure only: SBP ≥147 mm Hg, AR ≤15 %) had a range of reassessment responses from 2 to 3 days up to 5 years, with similar ranges in other categories. Previous research has also pointed to an evidence-practice gap in the uptake of AR assessment as a tool for primary CVD prevention, but reassessment has not specifically been investigated in this literature [19–21]. Although in this hypothetical study high risk patients would be recalled for CVD risk reassessment sooner, previous studies have shown that in practice many people who fall into this category are undertreated [20, 21].

However, the qualitative results reveal justifications for frequent monitoring and reassessment of CVD risk that are not necessarily accounted for in the guidelines, including the perceived benefits of more frequent monitoring and reassessment on patient motivation to make and maintain lifestyle changes. Perceived patient motivation can be used as a rationale for both frequent and infrequent reassessment intervals. Additionally, several GPs mentioned the difficulty of convincing lower risk patients that they do not require frequent blood tests and reassessment. In the experimental study, GPs also made comments about recalling patients for non-CVD related matters even though the question was posed in the context of CVD risk assessment. This may reflect how GPs view CVD risk in the context of their patient’s overall health and the broad spectrum of risk factors that GPs are continuously monitoring and (re)assessing in their patients. These varied reasons for monitoring and reassessment, which take into account both biological and psychosocial patient factors, are not acknowledged in the guidelines and lead to wide practice variability. More guidance is needed for GPs around when to perform CVD risk reassessment to inform decision-making versus monitoring risk factors in order to fulfil other psychosocial functions. It should be noted that while the experiment presented here deliberately removed contextual patient factors such as preferences and other health concerns, these are important considerations for patient-centred practice.

The strengths of this study include 1) a heterogeneous sample and 2) the use of both quantitative and qualitative methods in order to enhance understanding of GPs’ decision-making regarding reassessment of primary CVD risk. The main limitations are that 1) GPs who participated in the study may be more or less supportive of using AR assessment than GPs in the wider community, 2) the study relied on self-reported behaviour, which may differ from actual practice, and 3) GPs in the experimental study were asked “When would you reassess this patient?”, so it is possible that for patients who were prescribed a blood pressure or cholesterol lowering medication, GPs answered this question in terms of treatment monitoring, rather than CVD risk reassessment. However, this does not change the key finding that the majority of low risk patients, who should not be prescribed medication according to the guidelines [1], were reassessed much earlier than recent modelling recommends: less than 1 year compared to 8–10 years [6].

## Conclusions

There is a lack of evidence-based guidance for reassessing primary CVD risk, and as these studies show, what GPs do in practice is very inconsistent. Many are recalling patients too frequently, but the reasons reflect a broader view of patients’ health, incorporating psychosocial factors. The use of shorter intervals for low-moderate AR contradicts research on optimal monitoring intervals [6], and may result in unnecessary costs and over-treatment. Evidence on when to reassess patients’ primary CVD risk needs to be better integrated into clinical practice guidelines, while also acknowledging other legitimate reasons for more frequent monitoring.

## Abbreviations

AR, absolute risk; BMI, body mass index; CVD, cardiovascular disease; GEE, generalised estimation equation; GP, general practitioner; HDL, high-density lipoprotein; IR, individual risk; SBP, systolic blood pressure; TC, total cholesterol

## Additional file

Additional file 1: Study 2 qualitative interview questions about how GPs reassess primary CVD risk. (DOCX 14 kb)
