# Supplementary material for: Factors influencing general practitioners’ decisions about cardiovascular disease risk reassessment: findings from experimental and interview studies
Source: BMC Fam Pract. 2016 Aug 5;17:107. doi: 10.1186/s12875-016-0499-7 (PMC4974805; doi:10.1186/s12875-016-0499-7)
Supplement: Additional file 1: — Study 2 qualitative interview questions about how GPs reassess primary CVD risk. (DOCX 14 kb) [file 12875_2016_499_MOESM1_ESM.docx]

**Factors influencing General Practitioners’ decisions about cardiovascular disease risk reassessment: Findings from experimental and interview studies - Appendix 1**

**Expectations and experiences of re-assessment**

1. How do you manage patients who are at elevated risk of cardiovascular disease, but the risk isn’t high enough to start medical treatment straight away?
2. If you re-assess them,
   1. How do you reassess them?
   2. How frequently do you reassess them?
   3. What factors influence what you do and how often you do it?
3. What do you tell your patients when recommending re-assessment?
4. What are your main aims of re-assessment?
5. Are there any aspects of re-assessment that can be difficult to manage or concern you?
6. From your experience, what would you say are the main things the patients want from coming to see you for re-assessment?
   1. Do they ever report feeling worried prior to their appointments or seem anxious while here?
   2. How do you manage patient anxiety or concerns?
   3. Do they report feeling relieved or reassured?
7. Once your patients are on medical treatment, how do you monitor them?
